# Mosquito diversity and dog heartworm prevalence in suburban areas

**DOI:** 10.1186/s13071-019-3874-0

**Published:** 2020-01-10

**Authors:** Meredith R. Spence Beaulieu, Jennifer L. Federico, Michael H. Reiskind

**Affiliations:** 1 Department of Entomology and Plant Pathology, North Carolina State University, Raleigh, NC USA; 2 Animal Services Division, Department of Environmental Services, Wake County Animal Center, Raleigh, NC USA

**Keywords:** Mosquito, Heartworm, Urbanization, Diversity, Vector, Landscape, Disease ecology

## Abstract

**Background:**

Urbanization is occurring rapidly on a global scale and is altering mosquito communities, creating assemblages that are characteristically less diverse. Despite high rates of urbanization and ample examples of vector-borne diseases transmitted by multiple species, the effects of urbanization-driven mosquito diversity losses on disease transmission has not been well explored. We investigated this question using the dog heartworm, a filarial parasite vectored by numerous mosquito species.

**Methods:**

We trapped host-seeking mosquitoes in undeveloped areas and neighborhoods of different ages in Wake County, North Carolina, USA, analyzing captured mosquitoes for heartworm DNA. We compared within-mosquito heartworm infection across land-use types by Kruskal–Wallis and likelihood ratio tests. Using zip code level data acquired from dogs in a local shelter, we performed linear regressions of within-host heartworm prevalence by within-mosquito heartworm prevalence as well as by three mosquito diversity measures. We also determined the best predictor of host-level prevalence among models including within-mosquito infection, mosquito diversity and abundance, and socioeconomic status as variables.

**Results:**

Suburban areas had lower within-mosquito heartworm prevalence and lower likelihood of heartworm-positive mosquitoes than did undeveloped field sites, although no differences were seen between suburban and undeveloped wooded sites. No relationships were noted between within-mosquito and within-host heartworm prevalence. However, mosquito diversity metrics were positively correlated with host heartworm prevalence. Model selection revealed within-host prevalence was best predicted by a positive relationship with mosquito Shannon–Wiener diversity and a negative relationship with household income.

**Conclusions:**

Our results demonstrate that decreases in mosquito diversity due to urbanization alter vector-borne disease risk. With regard to dog heartworm disease, this loss of mosquito diversity is associated with decreased heartworm prevalence within both the vector and the host. Although the response is likely different for diseases transmitted by one or few species, mosquito diversity losses leading to decreased transmission could be generalizable to other pathogens with multiple vectors. This study contributes to better understanding of the effects of urbanization and the role of vector diversity in multi-vectored pathosystems.
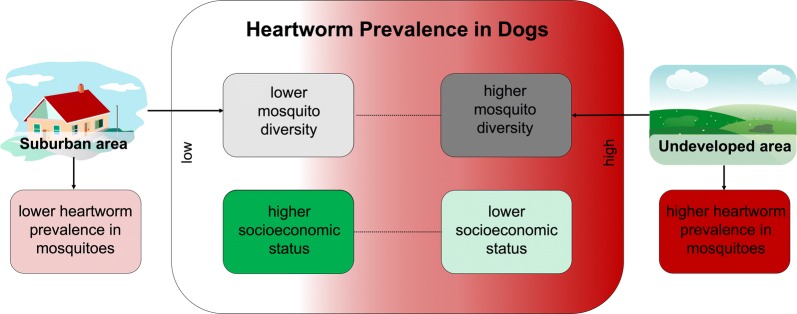

## Background

Mosquitoes are a diverse family of flies that can be pests and vectors of medical and veterinary significance, transmitting a wide array of viruses, protozoans and parasitic nematodes to both humans and animals. Many important pathogens are transmitted by a limited number of mosquito vectors, and both research and control efforts have focused on targeting these primary vectors. However, there are numerous examples of disease systems in which an assembly of vector species are responsible for pathogen transmission (e.g. [1–4]). The focus of studies on primary vectors rather than communities of vectors has led to a general gap in knowledge as to how vector diversity contributes to disease transmission in multi-vectored pathosystems, even though the limited empirical and modeling investigations that have occurred suggest vector diversity can increase transmission [5, 6]. Given that anthropogenic land-use change alters the diversity and composition of mosquito assemblages and is occurring rapidly worldwide, greater understanding of the role of vector diversity in multi-vectored diseases is critical and will have global impacts.

The effect of urbanization is well known for certain mosquito species of interest, particularly the container-breeding *Aedes* [7, 8], but has only recently been examined in the context of effects on mosquito species assemblages. Urbanized areas tend to have distinct mosquito communities that are characteristically less diverse than those in natural habitats [9–13]. At least in the context of suburban areas, mosquito communities do not recover from these diversity losses after the initial land-use change; diversity decreases as neighborhoods age, resulting in the lowest diversity mosquito assemblages in the most established suburban areas [10]. For both suburban and urban areas generally, anthropogenic disturbance is also associated with increased abundance of vectors of human disease, including container-breeding *Aedes* that transmit dengue, Zika, or chikungunya viruses, and *Culex* mosquitoes, which transmit West Nile virus and human filarial pathogens [10, 11, 13, 14]. The shift in mosquito assemblage to a lower richness community composed of a high proportion of known vectors likely increases disease transmission for most pathogens [9, 15]. However, other studies have suggested that for diseases transmitted by a variety of vectors, such as malaria or hemorrhagic disease in deer, vector species richness is strongly positively correlated with disease prevalence, possibly due to functional diversity extending the transmission season [5, 16]. An ideal system for further examining the effects of mosquito diversity on vector-borne disease transmission is that of the dog heartworm, *Dirofilaria immitis*.

The nematode *D. immitis* is an obligate parasite of mosquitoes and canids. Mosquitoes acquire microfilaria, the mosquito-infective parasite stage, upon ingestion of a blood meal from an infectious canine host. The parasite develops through multiple larval stages within the mosquito, culminating in the host-infective third larval stage (L3), which enters the bite wound of a susceptible host during the mosquito’s next blood-feeding. Within the canine host, the adult heartworm resides in the pulmonary arteries and the heart, causing respiratory distress and eventually congestive heart failure [17]. Dog heartworm disease is global in distribution and is likely the most common vector-borne disease in the USA, with prevalence in domestic dogs between 1–12.5% on average nationwide [17], but as high as 48.8% in certain highly endemic regions following natural disasters, like the Gulf Coast post-Hurricane Katrina [18]. Dog heartworm is considered endemic in the contiguous USA, with highest prevalence in the southeastern USA [19]. Importantly, the parasite is naturally vectored by at least 25 mosquito species in the USA [20] and even greater numbers of species worldwide with varying vectorial capacities.

As dog heartworm disease is vectored by an assemblage of mosquito species, changes in mosquito diversity will likely affect disease prevalence. Urbanization is one context in which vector assemblage and diversity vary, potentially with implications for heartworm disease transmission. While a previous study has investigated dog heartworm prevalence within mosquitoes in an urban to rural gradient [21], the effects of urbanization-driven mosquito diversity changes on the pathosystem have not been previously considered. Urban and suburban areas throughout much of the USA are dominated by the peridomestic *Aedes albopictus*, which is a competent vector of *D. immitis* [20]. It is possible that heartworm disease risk could be higher in urbanized areas [21], where the majority of mosquito bites are likely to be from *Ae. albopictus*, *versus* in natural areas, where bites come from a more diverse mosquito assemblage in which a given species may or may not be a competent *D. immitis* vector. However, with vector diversity being linked to both increased and decreased disease transmission depending on the pathosystem, *D. immitis* risk as a function of urbanization-induced vector diversity changes is difficult to predict.

Although vector biodiversity is our primary interest, another potentially important contributor to dog heartworm disease risk is socioeconomic status. There is effective preventative medication that protects dogs from becoming infected with *D. immitis*, but these medications are by prescription only, requiring not only the cost of the medication but also the cost of routine veterinary care, presenting a financial barrier for some pet owners. As expected, people of higher socioeconomic status reported greater use of preventative medications, resulting in lower levels of vector-borne pathogens, including *D. immitis* [22]. Additionally, lower socioeconomic status could increase disease risk *via* increased vector exposure, either through behavioral factors (e.g. dogs spending more time outdoors) or through changes in vector assemblages. Studies investigating socioeconomic gradients have variably shown greater mosquito prevalence in lower income neighborhoods [23, 24] and in higher income neighborhoods [25], as well as no effect on mosquito densities [26]. While the directionality of its relationship with mosquito communities remains unclear, it is evident that socioeconomic status has the ability to alter susceptibility of hosts, vector exposure, and therefore disease risk.

In the present study, we sought to determine the relationship between mosquito diversity and *D. immitis* prevalence in domestic dogs within the suburban setting. We approached this question by sampling mosquitoes across Wake County, North Carolina, USA, analyzing the mosquitoes for the presence of *D. immitis* DNA, and comparing heartworm prevalence rates within the mosquito to heartworm prevalence rates within domestic dogs. Recognizing that mosquito diversity changes as suburban neighborhoods grow older [10], we stratified our mosquito sampling efforts in suburban areas by neighborhood age, creating a chronosequence with which to test our predictions. Based on the results of a prior study of heartworm prevalence on an urban to rural gradient [21], we hypothesize that older neighborhoods with less diverse mosquito assemblages dominated by *Ae. albopictus* have greater dog heartworm prevalence. We also considered that socioeconomic status may affect disease prevalence, hypothesizing that higher income areas have less dog heartworm than low income areas.

## Methods

### Mosquito sampling

Mosquitoes used for analysis in this study were previously sampled and the effects of suburban development on mosquito diversity were previously assessed in Spence Beaulieu et al. [10]. Briefly, we identified suburban neighborhoods of various ages throughout Wake County, NC, USA using Google Earth historical images [27], taking the year of first evidence of construction as the year the neighborhood was built. We created categories of neighborhood ages to ensure that neighborhoods of various ages were being sampled: developed before 1993, between 1993–2002, between 2003–2007, between 2008–2012 and from 2013 to present. Overall, we selected 30 neighborhoods in 2015 that were constructed within the last 40 years and added an additional 6 older neighborhoods in 2016 that were developed between 50 and 102 years prior. For each neighborhood, we trapped at a single house with homeowner approval, verifying that homeowners did not intend on doing any mosquito barrier sprays or insecticidal treatments during the study period.

In addition to suburban sites, we sampled in three natural habitat sites: Schenck Memorial Forest at North Carolina State University (NC State), NC State’s Equine Educational Unit and NC State’s Lake Wheeler Beef Unit. Each of these sites contained some wooded habitat and some field or pasture habitat, allowing us to conveniently sample mosquitoes in these two distinct habitat types. We also sampled at 6 additional smaller parcels of land composed of undeveloped woodlots and 5 additional smaller parcels of land composed of undeveloped fields. Each of these smaller natural sites had a radius of at least 100 m of undeveloped land around the trap, which is an appropriate radius given previous findings on mosquito habitat fidelity [28]. Overall, we trapped at a total of 9 wooded sites and 8 field sites throughout Wake County.

At each of the suburban and undeveloped sites, we sampled overnight with CDC light traps (JW Hock Co., Gainesville, FL, USA) baited with 1 kg of dry ice (solid CO_2_). Lights were removed from the CDC light traps to reduce by-catch. In the suburban neighborhoods, trap placement within the yard was based on the homeowner’s preference. At the wooded and field sites, traps were placed at least 100 m away from any habitat edge, again consistent with previous findings of mosquito habitat fidelity [28]. We trapped at each site biweekly from June through mid-October in 2015, and June through the end of October in 2016. We identified all mosquitoes to species using published dichotomous keys [29, 30].

### Molecular analysis

For mosquitoes collected in 2015, we sexed the individuals and females were dissected for parity analysis *via* ovary tracheation [31]. Because of their inability to be infectious for *D. immitis*, we excluded males and nulliparous females from further analysis. We then pooled parous females by site, date collected and species for molecular analysis, with up to 19 individual mosquitoes per pool. We did not dissect mosquitoes collected in the 2016 trapping season, but rather immediately pooled all female mosquitoes by site, date collected and species, again with up to 19 individual mosquitoes per pool.

In a separate facility, we extracted DNA from each pool using either the DNeasy Blood and Tissue Kit (Qiagen, Venlo, Netherlands) or the ZR Genomic DNA-Tissue MiniPrep (Zymo Research, Irvine, CA, USA). We homogenized the mosquitoes using sterilized pestles in sterilized microcentrifuge tubes and followed modified versions of the standard kit protocols for DNA extraction (full extraction protocols are presented in Additional file 1: Text S1). After extraction, we quantified the DNA concentration using the Qubit 2.0 Fluorometer (Invitrogen, Life Technologies Corporation, Waltham, MA, USA), accepting any sample with greater than 0.01 ng/μl of DNA for further analysis. We stored the extracted DNA at − 20 °C until the time of further analysis.

We performed real time quantitative polymerase chain reaction (qPCR) to assess for presence or absence of *D. immitis* DNA within the previously extracted and quantified mosquito pools. To minimize the risk of sample contamination, qPCR was performed at a dedicated workstation that was separate from that utilized for DNA extraction. Each qPCR reaction consisted of 0.2 µl each of an established *D. immitis*-specific COI forward and reverse primer (DI COI primer pair) [32], 5 µl of SYBR green master mix, 2 µl of template DNA and 2.6 µl of water for a total reaction volume of 10 µl. Each 96-well plate run included one reaction with DNA extracted from a known negative laboratory-reared mosquito combined with a single laboratory-reared *D. immitis* L3 for the dual purpose of both a positive control and a sensitivity check to ensure *D. immitis* could be detected down to the presence of a single larva. Additionally, each 96-well plate included both a reaction with no template and a reaction with DNA extracted from a known negative laboratory-reared mosquito as negative controls. All samples and controls were run in duplicates to ensure accuracy of results. The qPCR procedure consisted of a denaturation step at 95 °C for 30 s, followed by 40 cycles of denaturation at 95 °C for 5 s, annealing at 60 °C for 15 s and extension at 72 °C for 10 seconds. Because the goal was to determine simple presence or absence of *D. immitis* within the mosquito pools, CFX Maestro software (Bio-Rad Laboratories, Hercules, CA, USA) was used to visually assess for DNA amplification and therefore presence of *D. immitis* DNA within the sample prior to 30 cycles.

To verify that qPCR pools were positive for *D. immitis*, all positive amplicons were submitted to Eton Bioscience (San Diego, CA, USA) for Sanger sequencing. Sequences were assembled using the Geneious 9.1.8 native *de novo* assembly algorithm and consensus sequences were generated for each sample. As all sample sequences were identical based on a reference alignment, one sample sequence was used in the MegaBLAST algorithm from the NCBI Nucleotides BLAST suite, returning a positive match to a portion of *D. immitis* COI with 100% homology (GenBank: AJ537512.1) [33].

### Within-host heartworm data acquisition

We acquired data from the Wake County Animal Center on all dogs entering the shelter either as an owner surrender or as a captured stray between January 2010 and October 2015. These data contained the results of the heartworm test performed at the time of intake as well as the zip code of the prior owner or location of capture. Any dogs without heartworm test results or a designated zip code were removed from analysis.

### Statistical analyses

For each mosquito species, we calculated total number of individuals and total number of pools analyzed, the number of pools positive for *D. immitis*, and the percent *D. immitis* positive pools. We calculated the bias corrected maximum likelihood estimate (MLE) for point estimation of the infection rate of each heartworm-positive mosquito species using PooledInfRate add-in software for Excel [34]. We investigated seasonality of *D. immitis* transmission within Wake County by plotting the overall percent positive pools across the sampling season. Since parity data for mosquitoes were collected only in 2015, we assessed for correlation of parous mosquitoes with heartworm-positive pools in 2015.

We performed two Kruskal–Wallis tests to compare heartworm prevalence across land-use types in R 3.5.0 statistical software [35]. Kruskal–Wallis test was chosen due to the non-normal nature of the data, and Dunnʼs test for multiple comparisons with Bonferroni correction was utilized to identify which land-uses differed in their heartworm prevalence. The first test compared heartworm prevalence between suburban, natural woodlot and natural field sites with all neighborhood ages collapsed into a single suburban category (three total treatment levels). The second test included neighborhood age categories separately, resulting in seven total treatment levels (wood, field and five neighborhood age categories as previously described). To further investigate differences between land-use types while accounting for varying mosquito pool sizes during our molecular analysis, we used a likelihood ratio test to compare probability of within-mosquito *D. immitis* infection across land-use types. We maximized the likelihood function:

$$L\left( {p_{i} } \right) = \left( {\left( {1 - p_{i} } \right)^{{n_{i\,j} }} } \right)^{{\left( {1 - y_{i\,j} } \right)}} *\left( {1 - \left( {1 - p_{i} } \right)^{{n_{i\,j} }} } \right)^{{y_{i\,j} }}$$

where $y_{i\,j}$ is the binary response of whether pool $j$ in habitat $i$ was positive for *D. immitis*, $n_{i\,j}$ is the number of mosquitoes in pool $j$ from habitat $i$, and $p_{i}$ is the probability that an individual mosquito in habitat $i$ is positive for *D. immitis*. We then tested the null hypothesis that $\sum p_{i} = p$ using a likelihood ratio test, utilizing the Holm method to correct for multiple comparisons when identifying which land-uses differed in their probabilities. As with the Kruskal–Wallis test, we again performed this test for land-use type both with and without neighborhood age categories as levels within the broader suburban category.

We calculated heartworm prevalence within dogs by zip code in Wake County. We then calculated proportion heartworm-positive mosquito pools by zip code so that the two datasets were at comparable scales. We created choropleths to visualize risk maps for *D. immitis* infection within dogs and within mosquitoes in the *choroplethrZip* package in R [36]. We performed a linear regression of within-mosquito heartworm prevalence *versus* within-host heartworm prevalence. This approach sought to assess whether the heartworm status of mosquitoes was a reliable predictor of infection status within the host.

We calculated mosquito rarefied richness, Pielou’s evenness and Shannon–Wiener diversity as previously described [10] and found the average for a given zip code, then performed linear regressions comparing these diversity measures to within-host heartworm prevalence, testing the hypothesis that mosquito diversity impacts heartworm disease transmission. To disentangle the effects of mosquito diversity and mosquito abundance, we also calculated the average abundance within a zip code per site per trap-night and performed a linear regression comparing log average mosquito abundance to within-host heartworm prevalence. We acquired median household income by zip code from the 2013–2017 American Community Survey using U.S. Census Bureau’s American FactFinder tool [37] to investigate whether *D. immitis* prevalence within dogs and socioeconomic status is correlated. Finally, we performed generalized linear model selection to find the model that best explained within-host heartworm prevalence using all combinations of the following independent variables at the zip code level: presence or absence of heartworm-positive mosquito pools, proportion heartworm-positive mosquito pools, rarefied richness, evenness, Shannon–Wiener diversity, mosquito abundance and median household income. We performed a logit transformation of our within-host heartworm prevalence data to account for its proportional nature [38], and used Akaike information criterion (AIC) as the estimator of model quality in our model selection.

## Results

We collected a total of 10,244 mosquitoes over the two years of sampling. The most prevalent species was *Ae. albopictus*, the Asian tiger mosquito, which constituted 41.9% of the overall abundance, followed by *Culex salinarius* and *Aedes vexans* with 13.9% and 8.3%, respectively. After excluding males and nulliparous females as previously described, 8483 individuals in 2488 pools were tested for the presence of *D. immitis* DNA.

**Fig. 1:**
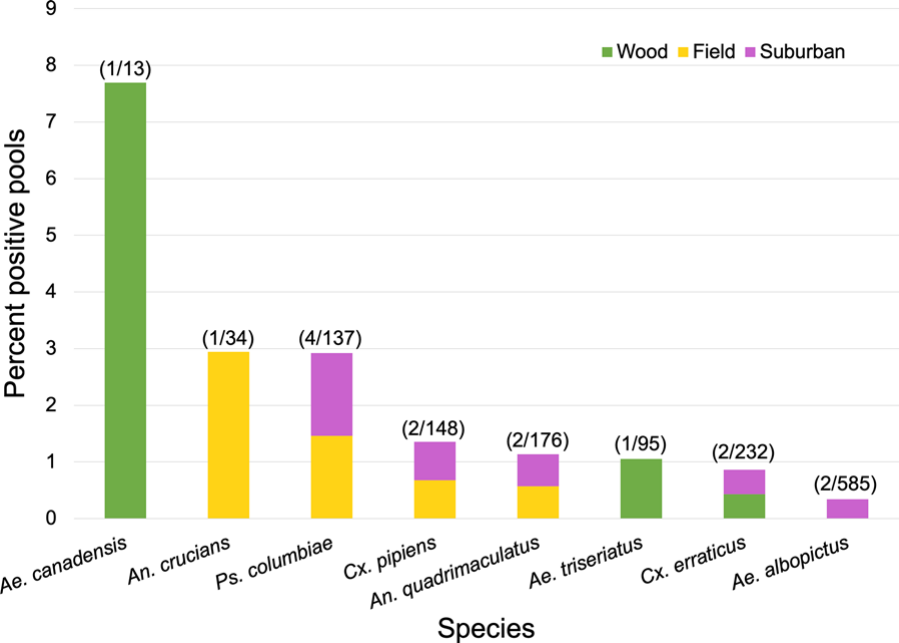
Species-level heartworm prevalence by land-use type. Eight mosquito species had pools that tested positive for *Dirofilaria immitis*. Percent positive pools by species is presented, with bars color-coded to denote the land-use type where the positive pool originated. Since sample sizes varied among species, the number of positive pools and the total number of pools tested per species are provided above each bar

**Fig. 2:**
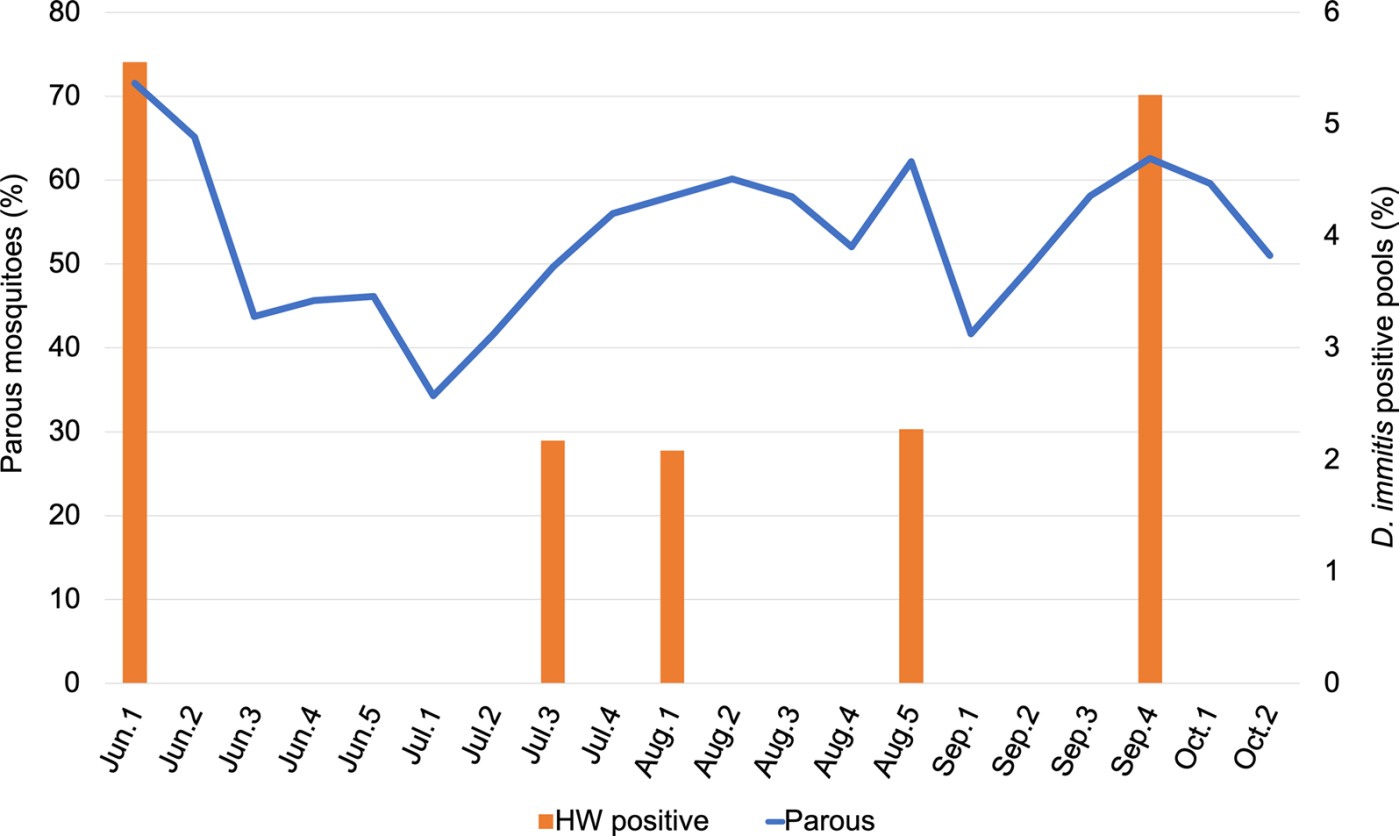
Relationship between mosquito parity and within-mosquito heartworm prevalence. Percent parous mosquitoes and percent *Dirofilaria immitis*-positive mosquito pools were compared for data from the 2015 trapping season. The two variables were positively correlated (Spearman’s *ρ *= 0.494, *P *= 0.027)

Of the total 2488 pools tested, 15 were positive for the presence of *D. immitis* DNA (Table 1). Positive pools were confirmed *via* sequencing, with all pools aligning to a portion of the *D. immitis* mitochondrion [33]. Eight mosquito species showed evidence of *D. immitis* infection, with *Aedes canadensis* having the highest percentage positive pools at 7.7% and the highest MLE infection rate at 29.75 per 1000 individuals (95% CI: 1.79–136), followed by *Anopheles crucians* and *Psorophora columbiae* both with approximately 2.9% positive pools and MLEs of 26.23/1000 (95% CI: 1.52–119.72) and 11.85/1000 (95% CI: 3.89–28.05), respectively (Table 1, Fig. 1). All other mosquito species had less than 1.4% *D. immitis-*positive pools and MLEs of less than 7.1/1000. The earliest *D. immitis-*positive pool of mosquitoes was collected during the first week of June, which coincided with the beginning of our trapping season, and the latest *D. immitis-*positive pool of mosquitoes was collected during the third week of October. No apparent seasonal trends in *D. immitis-*positive status were noted within the mosquito trapping season (see Additional file 2: Figure S1). However, when analyzing the 2015 data, percent heartworm-positive mosquito pools was positively correlated with percent parous mosquitoes across the trapping season (Fig. 2, Spearman’s *ρ *= 0.494, *P *= 0.027). As detailed in the Methods section, parity data were not collected in 2016 and correlation with heartworm-positive pools was therefore unable to be assessed for the second trapping year.

**Table 1:** Heartworm prevalence within mosquito species

Species	No. of individuals	No. of pools	Positive pools (land-use type)	Positive pools (%)	MLE (95% CI)
*Aedes albopictus*	3429	585	2 (suburban)	0.342	0.58 (0.1–1.91)
*Ae. atlanticus*	437	63	0	0	–
*Ae. canadensis*	33	13	1 (wood)	7.692	29.75 (1.79–136.00)
*Ae. cinereus*	3	3	0	0	–
*Ae. fulvus pallens*	1	1	0	0	–
*Ae. infirmatus*	13	7	0	0	–
*Ae. triseriatus*	182	95	1 (wood)	1.053	5.47 (0.32–26.14)
*Ae. vexans*	732	296	0	0	–
*Anopheles crucians*	38	34	1 (field)	2.941	26.23 (1.52–119.72)
*An. punctipennis*	377	198	0	0	–
*An. quadrimaculatus*	283	176	2 (1 field, 1 suburban)	1.136	7.05 (1.26–22.79)
*Coquillettidia perturbans*	63	41	0	0	–
*Culex* sp.^a^	6	6	0	0	–
*Cx. erraticus*	578	232	2 (1 wood, 1 suburban)	0.862	3.5 (0.62–11.48)
*Cx. peccator*	1	1	0	0	–
*Cx. pipiens*	340	148	2 (1 field, 1 suburban)	1.351	5.92 (1.06–19.26)
*Cx. restuans*	1	1	0	0	–
*Cx. salinarius*	1265	349	0	0	–
*Orthopodomyia signifera*	1	1	0	0	–
*Psorophora ciliata*	4	3	0	0	–
*Ps. columbiae*	339	137	4 (2 field, 2 suburban)	2.920	11.85 (3.89–28.05)
*Ps. ferox*	357	98	0	0	–
Total	8483	2488	15	0.603	1.77 (1.03–2.85)

^a^Too damaged to identify beyond genus level

*Notes*: For each mosquito species, total number of individuals captured throughout the study period, number of pools tested for the presence of *Dirofilaria immitis* DNA, number of positive pools, percent positive pools and maximum likelihood estimate (MLE) are reported. The land-use types from which the positive pools were collected are denoted in parentheses after the total number of positive pools by species. MLE values give a point estimate of the infection rate per 1000 individuals

**Fig. 3:**
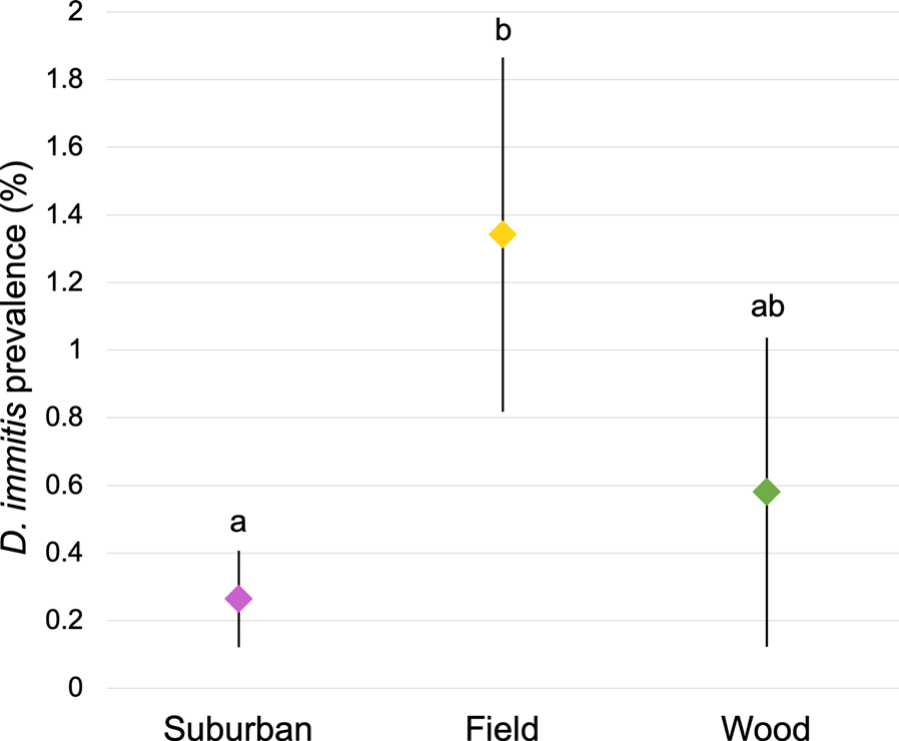
Comparison of within-mosquito heartworm prevalence by land-use type. Within-mosquito *Dirofilaria immitis* prevalence varied by land-use type (Kruskal–Wallis *χ*^2^= 8.555, *df *= 2, *P *= 0.014). Suburban sites had significantly lower *D. immitis* prevalence than did undeveloped field sites (*Z *= 2.925, *P *= 0.010). The prevalence in undeveloped wooded sites did not significantly differ from that in either suburban (*Z *= − 0.630, *P *= 1.0) or field sites (*Z *= 1.884, *P *= 0.179). Mean prevalence and standard error of the mean are presented for each land-use type. Letters above the bars denote significant differences

We found significant differences in within-mosquito *D. immitis* prevalence among land-use types (Kruskal–Wallis test: *χ*^2 ^= 8.555, *df *= 2, *P *= 0.014). Field sites had greater *D. immitis* prevalence than did suburban sites (Dunnʼs test: *Z *= 2.925, *P *= 0.010), but prevalence at wooded sites did not differ from that at suburban sites (Dunnʼs test: *Z *= − 0.630, *P *= 1.0) (Fig. 3). Neighborhood age was not a significant factor, as Kruskal–Wallis test was not significant when incorporating neighborhood age categories. We also found that the probability of an individual mosquito being positive for *D. immitis* differed between land-uses (LRT statistic = 6.40, *P *= 0.041). Field sites had greater likelihood of a mosquito being positive for *D. immitis* than did suburban sites (LRT statistic = 5.81, Holm adjusted *P *= 0.048), but the probability at wooded sites did not differ from that at suburban sites (LRT statistic = 0.013, Holm adjusted *P *= 0.911) or field sites (LRT statistic = 4.99, Holm adjusted *P *= 0.051). Again, neighborhood age was not a significant factor, as the likelihood ratio test was not significant when incorporating neighborhood age categories into the analysis.

**Fig. 4:**
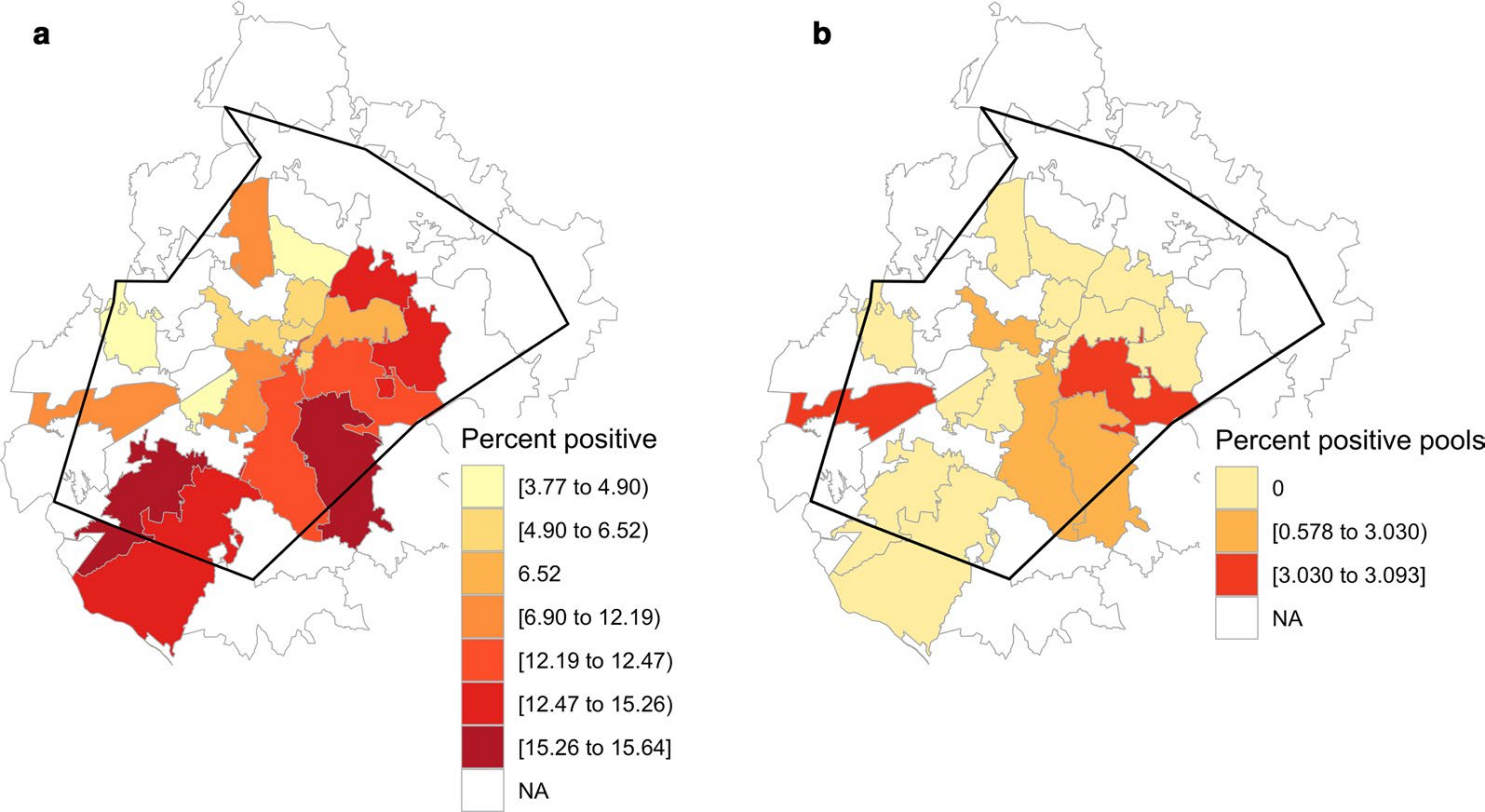
Visualization of heartworm prevalence by zip code in Wake County, North Carolina. **a** Heartworm prevalence within dogs ranged from 3.77% to 15.64% within zip codes where mosquitoes were sampled. All zip codes in Wake County had some level of heartworm infection, but zip codes where mosquitoes were not sampled were omitted from visualization (denoted NA) for clarity. **b** Heartworm prevalence within mosquitoes ranged from 0% to 3.09% by zip code. Due to low overall numbers of *Dirofilaria immitis-*positive mosquito pools across all trapping sites, many zip codes had no detected within-mosquito heartworm infection. Zip codes where mosquitoes were not sampled are denoted NA

A total of 7625 dogs were tested for heartworm and designated a zip code upon shelter intake. Of these, 832 tested positive for *D. immitis*, giving an average prevalence of 10.91% in domestic dogs in Wake County. A risk map for *D. immitis* infection within the canine host shows a trend of higher prevalence in eastern and southern Wake County zip codes (Fig. 4a). We cannot assess spatial trends for *D. immitis* within mosquitoes due to low overall prevalence (Fig. 4b). When comparing *D. immitis* prevalence within dogs to its prevalence within mosquitoes by zip code, no significant relationship was detected (*F*_(1, 16)_= 0.511, *P *= 0.485). Given that the majority of zip codes had a within-mosquito heartworm prevalence of 0% due to our low number of positive pools, we also performed a Welch’s t-test comparing the within-host heartworm prevalence by the presence or absence of heartworm-positive mosquito pools within a given zip code. We used this binary designation as an additional method to investigate any detectable relationships between vector infection and host infection in our dataset. Similar to our results with within-mosquito prevalence, using the presence or absence of *D. immitis* within mosquitoes, we did not see a significant relationship to within-host prevalence by zip code (*t*_(8.22)_= − 0.941, *P *= 0.374).

**Fig. 5:**
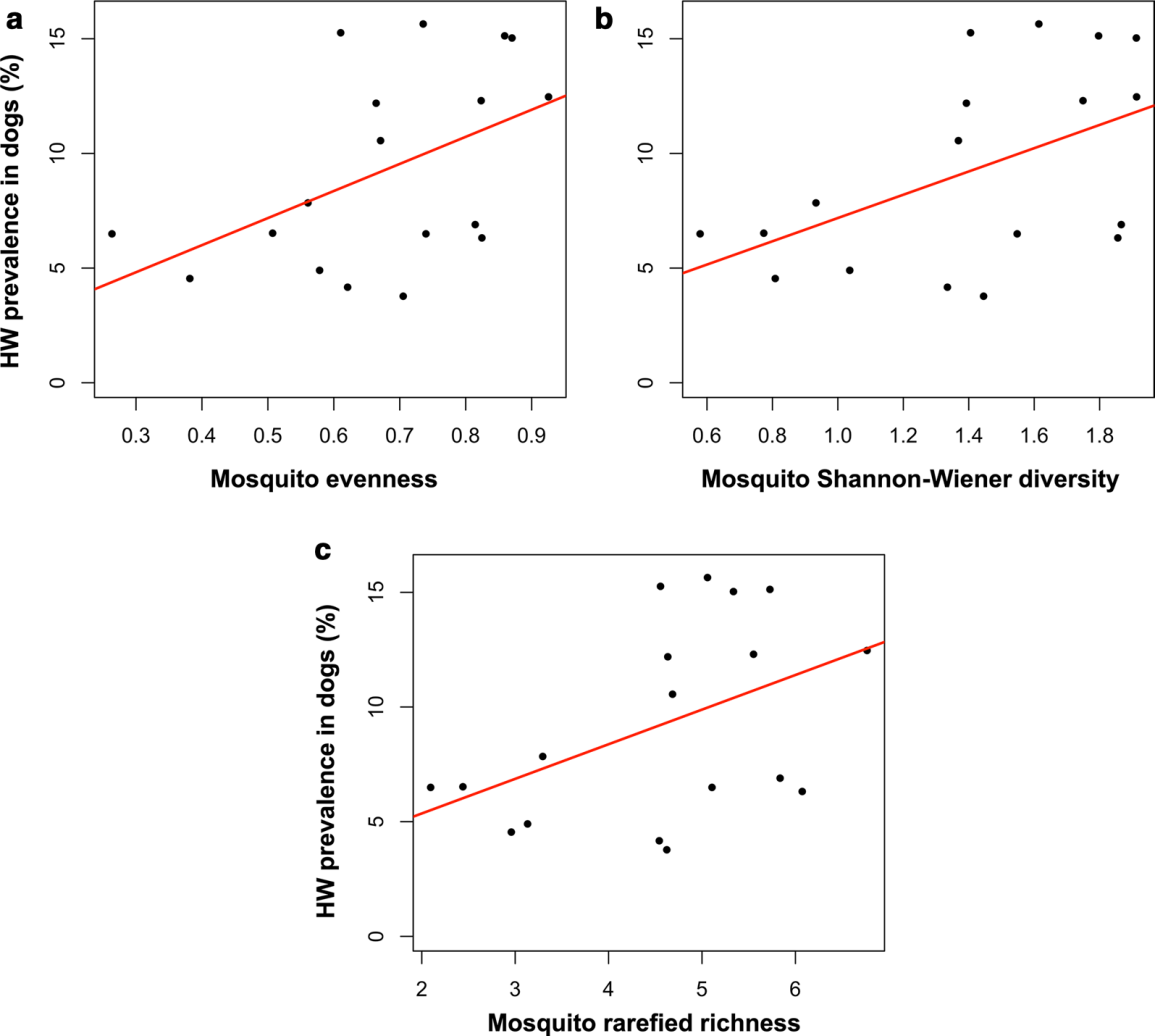
Within-host heartworm prevalence increases with mosquito diversity measures. A significant positive correlation was noted between within-host heartworm prevalence and (**a**) mosquito evenness (*F*_(1, 16)_= 4.881, *P *= 0.042, *R*^2^= 0.234) as well as (**b**) mosquito Shannon–Wiener diversity (*F*_(1, 16)_= 5.464, *P *= 0.033, *R*^2^= 0.255). **c** While the relationship between within-host heartworm prevalence and mosquito rarefied richness was not significant (*F*_(1, 16)_= 4.342, *P *= 0.054, *R*^2^= 0.213), a similar positive trend was found

When investigating correlations of mosquito diversity metrics at the zip code level to within-host *D. immitis* prevalence, we detected significant positive relationships for evenness (*F*_(1, 16)_= 4.881, *P *= 0.042, *R*^2^= 0.234) and Shannon–Wiener diversity (*F*_(1, 16)_= 5.464, *P *= 0.033, *R*^2^= 0.255) (Fig. 5a, b). While the relationship with rarefied richness was not significant (*F*_(1, 16)_= 4.342, *P *= 0.054, *R*^2^= 0.213), there was a similar positive trend (Fig. 5c). We did not find a relationship between log mosquito abundance and within-host heartworm prevalence (*F*_(1, 16)_= 0.396, *P *= 0.538). Among models including all combinations of our tested variables (presence or absence of heartworm-positive mosquito pools, proportion heartworm-positive mosquito pools, rarefied richness, evenness, Shannon–Wiener diversity, mosquito abundance and median household income), selection revealed that the top model set (all models with ΔAIC < 2) included two models: (i) mosquito Shannon–Wiener diversity and median household income (*AIC *= 23.95, residual *df *= 15, *ΔAIC *= 0); and (ii) mosquito Shannon–Wiener diversity, mosquito rarefied richness and median household income (*AIC *= 25.45, residual *df *= 14, *ΔAIC *= 1.5). Since the latter model is a more complex version of the prior nested model that has greater AIC support [38], the model including only mosquito Shannon–Wiener diversity and median household income is the best predictor of *D. immitis* prevalence within the canine host. This model was significant, with prevalence positively correlated with diversity and negatively correlated with median household income (DogHWPrev= 0.6454 ShanDiv – 1.035 * 10^−5^ Income − 2.524; *F*_(2, 15)_= 6.725, *P *= 0.008, *R*^2^= 0.473).

## Discussion

We found that suburban areas generally had the lowest within-mosquito heartworm prevalence, and that mosquito diversity was positively correlated with heartworm prevalence within the canine host. Based upon the increased abundance of suspected vectors, we originally predicted that older neighborhoods with less diverse mosquito assemblages would have greater dog heartworm prevalence, but this was not supported by our findings. Instead, our results suggest a positive relationship between mosquito diversity and disease transmission, as has been found in the limited existing studies investigating the effects of diversity within disease systems with multiple vectors [5, 6, 16]. Within the landscape of North Carolina, this equates to less heartworm disease expected within the host in suburban areas as a function of urbanization-induced mosquito diversity losses. With regard to socioeconomic status, we found support for our prediction of a negative relationship with heartworm prevalence within the host.

We analyzed entire mosquito bodies for the presence of *D. immitis* DNA; because of this, we were unable to distinguish between infected and infectious mosquitoes. All pools that were positive for *D. immitis* DNA were those of mosquito species known to be competent heartworm vectors [20], so we assume that any positive mosquito pool represents potential transmission. In terms of percent *D. immitis-*positive mosquito pools, *Ae. canadensis* and *An. crucians* were implicated as two important local vectors. However, these species only had positive pools detected in undeveloped natural areas. The mosquitoes implicated as heartworm vectors within suburban areas in this study were *Ps. columbiae*, *Cx. pipiens*, *Anopheles quadrimaculatus*, *Culex erraticus* and *Ae. albopictus*. Although uneven sampling size between species makes it difficult to assess which species are important local vectors, *Ps. columbiae* appears to be a significant contributor to heartworm transmission in suburban Wake County, as it was the only species in the current study to have > 1% *D. immitis-*positive pools in suburban neighborhoods.

There did not appear to be any seasonal trends in *D. immitis* infection within mosquitoes, although we could have missed important dynamics in the spring due to our trapping season beginning in June. Seasonal trends could also have been obscured by the overall low frequency of heartworm-positive mosquito pools. Yet throughout the trapping season, percent *D. immitis*-positive pools was positively correlated with mosquito parity data. As the percentage of mosquitoes in the area that have previously laid eggs (and therefore previously taken a blood meal) increases, *D. immitis* presence in mosquitoes also increases, reaffirming that older mosquitoes are the most dangerous from a disease transmission standpoint due to their greater probability of prior pathogen exposure. With so many known heartworm vectors spanning across multiple genera, the apparent lack of seasonality coupled with a positive correlation with parity could be due to vector mosquitoes having different phenologies, making heartworm transmission potential a nearly constant risk throughout the warmer months in North Carolina.

Contrary to our initial hypothesis, when comparing the land-use types of suburban neighborhoods, undeveloped woodlots and undeveloped fields, we found that field areas had significantly higher prevalence and likelihood of heartworm-positive mosquitoes than did suburban areas. Given the lower probability of *D. immitis-*positive mosquitoes noted in suburban areas, our focus on sampling mosquitoes predominantly in suburbia could have resulted in lower overall within-mosquito prevalence rates than what has been reported in other studies sampling in more rural landscapes (e.g. [39]). We found that two mosquito diversity metrics were positively correlated with heartworm prevalence within dogs at the zip code level. Our previous work has shown that mosquito rarefied richness, evenness and Shannon–Wiener diversity is decreased in established suburban neighborhoods when compared with undeveloped natural areas [10]. Taken together with the present findings, this suggests that suburban development is decreasing mosquito diversity, and that the resultant decreased mosquito diversity is linked with lower heartworm disease prevalence. This agrees with findings from a recent study that demonstrated a negative correlation between human population size and within-host heartworm prevalence [40]. While we did not detect any differences in heartworm prevalence within mosquitoes based on neighborhood age, it could still be affecting prevalence within the host indirectly by decreasing mosquito diversity, as mosquito diversity metrics decrease as suburban neighborhoods age [10].

The association between decreased mosquito diversity and decreased heartworm prevalence exists despite the fact that the dominant mosquito species in the sampled suburban areas (e.g. *Ae. albopictus*, *Cx. salinarius*, *Ae. vexans*, *Cx. pipiens* and *An. quadrimaculatus*) are known to be competent heartworm vectors [20]. A similar study of *D. immitis* prevalence within mosquitoes in an urbanized area of Oklahoma, USA found that urban sites had significantly higher heartworm infection rates than rural sites and implicated *Ae. albopictus* as the area’s primary vector [21]. These results do not agree with the present study’s findings of suburban areas having lower heartworm infection rates than at least the undeveloped field sites, and of *Ae. albopictus* not being a primary heartworm vector. One possible explanation for this discrepancy is that vector competence within a single mosquito species is susceptible to selection and can vary among geographically distinct populations [20, 41]. Studies on the vector competence of *Ae. albopictus* populations in North Carolina are rare, but have suggested that it is likely not a suitable vector for *D. immitis* in North Carolina [42]. If the local population is indeed refractory to *D. immitis* infection, that could drive the observed decreased heartworm prevalence in suburban areas, as over 40% of our trapped mosquitoes were *Ae. albopictus*.

We found that the best model to predict heartworm prevalence within dogs at the zip code level is one that includes both mosquito Shannon–Wiener diversity and household income. While mosquito diversity had a positive relationship with host heartworm prevalence in the model, household income had a negative relationship with host heartworm prevalence, supporting our hypothesis that higher income areas would have less dog heartworm disease than lower income areas. Interestingly, our previous work in Wake County did not find any effect of socioeconomic status on mosquito diversity measures [10], so the effect of socioeconomic status detected in the present study is likely due to its impact on host-level factors. This could be due to increased preventative medication use in higher income areas [22], or to variation in other factors such as the amount of time a dog spends outside and therefore amount of potential mosquito exposure time. While no host-level factors were explicitly investigated as drivers of dog heartworm prevalence in the present study, these factors are potentially important to dog heartworm disease dynamics and should be addressed in future studies. Another gap in host data is accurate information on wild canid populations that could be serving as reservoirs of dog heartworm. It has been suggested that coyotes are the most significant heartworm reservoirs in North America, with prevalence between 6.5 and 71% nationwide [43] and approximately 47% in North Carolina [44]. Wild host densities are not assessed in this study, but could play an important role in the heartworm transmission dynamics for domestic dogs, particularly if wild hosts that typically serve as primary *D. immitis* reservoirs are excluded from highly urbanized areas.

In addition to unmeasured host factors, this study is limited by the spatial scale of the within-dog heartworm prevalence data that we acquired. The zip code of the surrendered or stray dog was noted at shelter intake, allowing analysis of trends at the scale of zip code level or larger. Mosquitoes show habitat fidelity at a much finer scale of less than 100 m [28], leading to a separation of geographical scale between mosquito-level factors and host-level factors that could be obscuring some trends. Additionally, the history of the surrendered dogs is largely unknown, including for relevant factors such as travel, prior preventative medication usage, or the surrendering owner’s socioeconomic status. It is not possible to definitively tell whether the dog acquired the heartworm infection within the zip code of its primary residence, nor is it possible to tell whether the positive heartworm test represents a new or chronic infection due to the long lifespan of the parasite within the host. Despite these limitations, shelter data presents a large, readily available dataset that generally cuts across various human demographics, including income and education levels [45], and is the best data currently available with which to test our predictions. Future studies could partner with local veterinarians to get finer scale host data, as collection of detailed travel history for newly heartworm-positive dogs would allow for more definitive mapping of spatial and temporal host-level incidence trends.

Our results demonstrate that anthropogenic land-use change alters vector-borne disease risk. In the context of dog heartworm disease, the losses in mosquito diversity seen with suburban development are associated with decreased *D. immitis* prevalence in both the vectors and the host. Previous work with malaria has demonstrated a similar positive relationship between mosquito species richness and disease prevalence [5], suggesting that mosquito diversity losses being linked with decreased disease transmission could be applicable to a variety of multi-vectored diseases. However, decreases in mosquito diversity after human-driven land-use change could be detrimental when considering other disease systems of concern that are vectored by few mosquito species, such as dengue, chikungunya and Zika with *Ae. albopictus* [46, 47]. Urban and suburban development is predicted to increase by greater than 100% over the next 50 years in the southeastern USA [48], in line with global trends of increasing urbanization. With suburban and urban development rapidly changing global landscapes and the variable nature of the response of vector-borne diseases to these land-use changes, our understanding of the connection between vector diversity and disease transmission will become increasingly pressing and warrants further investigation.

## Conclusions

Our findings contribute to an understanding of the local transmission dynamics of the prevalent and devastating dog heartworm parasite in suburban areas of North Carolina, USA. We found an overall decrease in heartworm disease within the vector in suburban areas and a positive correlation between heartworm disease within the host and mosquito diversity measures, which are lower in suburban areas than in undeveloped areas. Within-host heartworm prevalence was well modeled by mosquito diversity and household income, further underscoring the effect of mosquito diversity while also illustrating the importance of socioeconomic status, possibly due to differences in administration of preventative medications. To our knowledge, this study represents the first explicit investigation of the effects of urbanization-driven mosquito diversity changes on dog heartworm transmission within both the vector and the host. Our results suggest that decreases in mosquito diversity due to urbanization lead to decreases in dog heartworm prevalence. This information can be utilized to identify areas of high mosquito diversity that may be foci for heartworm transmission. More broadly, our findings can be generalized to other pathogens with multiple vectors, contributing to an understanding of the role of arthropod diversity in multi-vectored disease systems.

## Supplementary information

**Additional file 1: Text S1.** Modified DNA extraction protocols used with both the DNeasy Blood and Tissue Kit (Qiagen, Venlo, Netherlands) and the ZR Genomic DNA-Tissue MiniPrep (Zymo Research, Irvine, CA, USA).

**Additional file 2: Figure S1.** Within-mosquito heartworm prevalence throughout the trapping season. Percent of mosquito pools positive for *Dirofilaria immitis* DNA for each week in the study’s trapping season is depicted. Trapping occurred over two years, but both years were analyzed together to obtain a single average point estimate for each calendar week.

## Data Availability

The datasets analyzed during the present study are available in the Figshare repository: 10.6084/m9.figshare.9339302.v1.
