# Supplementary material for: Mosquito diversity and dog heartworm prevalence in suburban areas
Source: Parasit Vectors. 2020 Jan 10;13:12. doi: 10.1186/s13071-019-3874-0 (PMC6953185; doi:10.1186/s13071-019-3874-0)
Supplement: Supplementary file 1 — Additional file 1: Text S1. Modified DNA extraction protocols used with both the DNeasy Blood and Tissue Kit (Qiagen, Venlo, Netherlands) and the ZR Genomic DNA-Tissue MiniPrep (Zymo Research, Irvine, CA, USA). [file 13071_2019_3874_MOESM1_ESM.pdf]

Additional file 1: Text S1

Modified DNA extraction protocol used with the DNeasy Blood and Tissue Kit (Qiagen, Venlo, Netherlands)

1. Homogenized sample in 170μl Buffer ATL. Added 30μl Proteinase K, mixed by vortexing, and incubated approximately 60-72 hours at 56°C. Vortexed for 15 seconds before proceeding.
2. Followed steps 2-7 as written in the Quick-Start Protocol.
3. Eluted DNA by adding 50μl dH_2O, incubating at room temperature for 1 minute, and centrifuging at 8,000 rpm for 1 minute.
4. Repeated elution as outlined in the previous step for a total eluted volume of 100μl.

Modified DNA extraction protocol used with the ZR Genomic DNA-Tissue MiniPrep (Zymo Research, Irvine, CA, USA)

1. Homogenized sample in a solution of 50μl 2X Digestion Buffer, 50μl dH_2O, and 5μl Proteinase K.
2. Incubated approximately 36-48 hours at 55°C.
3. Added 250μl Genomic Lysis Buffer and vortexed. Centrifuged at 10,000 rpm for 1 minute.
4. Followed steps 4-6 as written in the Solid Tissue Protocol, using half of the listed reagent volume (i.e. 100μl DNA Pre-Wash Buffer in step 5 and 200μl g-DNA Wash Buffer in step 6). Transferred the spin column to a new microcentrifuge tube.
5. Eluted DNA by adding 25μl DNA Elution Buffer, incubating at room temperature for 2-5 minutes, and centrifuging at top speed for 30 seconds.
6. Repeated elution as outlined in the previous step for a total eluted volume of 50μl.
