# Supplementary figures and images for: Mosquito diversity and dog heartworm prevalence in suburban areas
Source: Parasit Vectors. 2020 Jan 10;13:12. doi: 10.1186/s13071-019-3874-0 (PMC6953185; doi:10.1186/s13071-019-3874-0)

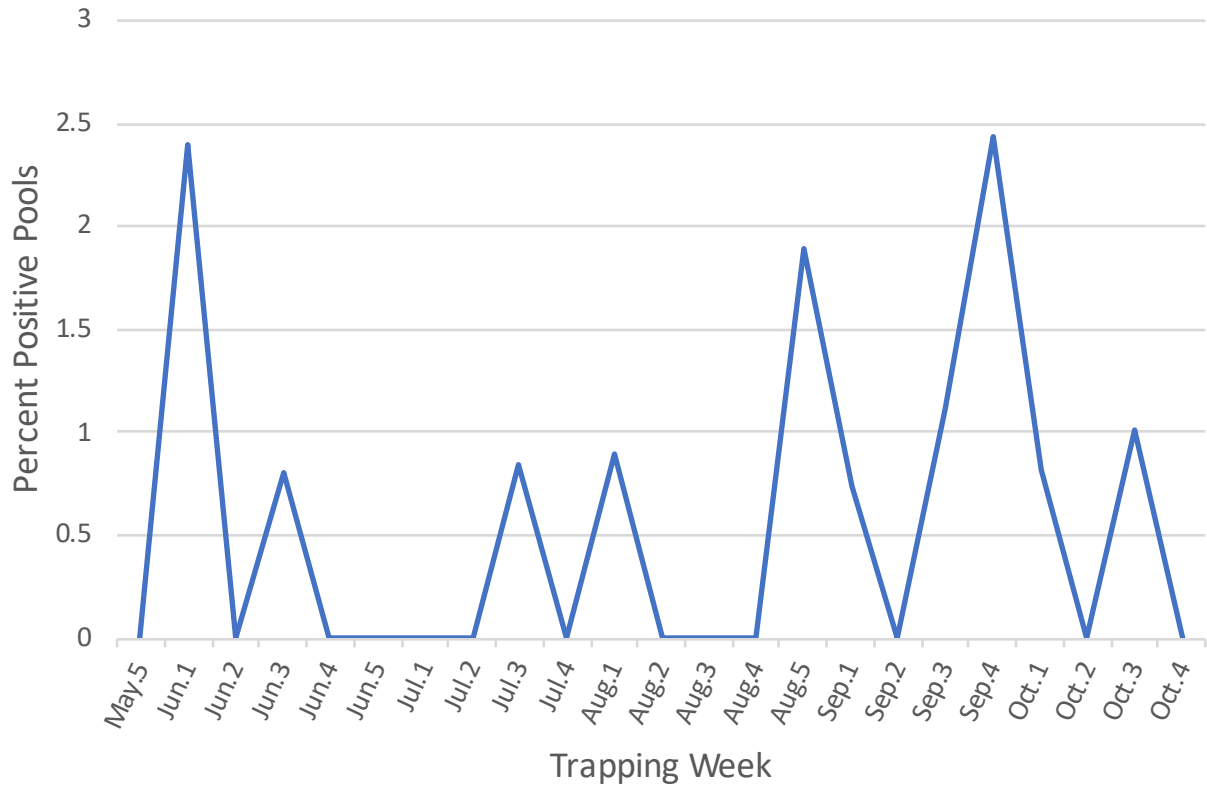

Supplement: Supplementary file 2 — Additional file 2: Figure S1. Within-mosquito heartworm prevalence throughout the trapping season. Percent of mosquito pools positive for Dirofilaria immitis DNA for each week in the study’s trapping season is depicted. Trapping occurred over two years, but both years were analyzed together to obtain a single average point estimate for each calendar week. [file 13071_2019_3874_MOESM2_ESM.pdf]
